# Impacts of hydrogeological characters of fractured rock on thermodynamic performance of ground-coupled heat pump

**DOI:** 10.1371/journal.pone.0252056

**Published:** 2021-05-26

**Authors:** Hang Zou, Peng Pei, Jin Zhang

**Affiliations:** 1 College of Mines, Guizhou University, Guiyang, Guizhou, China; 2 Institute of Groundwater and Earth Sciences, Jinan University, Guangzhou, Guangdong, China; China University of Mining and Technology, CHINA

## Abstract

Ground-coupled heat pump (GCHP) is used to recovery shallow geothermal energy, a widely distributed green energy source. Due to the imbalance between heat rejection and extraction, heat buildup underground is commonly associated with the long-term operation of GCHPs, which undermine system performance. Heat buildup intrinsically results the irreversibilities (entropy production) in subsurface heat sink, in which thermodynamic and transport properties are largely influenced by hydrogeologic properties, especially the existence of fractures and groundwater. This study investigates the influence of water flow in fractures on the thermodynamic performance of a single borehole heat exchanger (BHX) and heat buildup in the underground heat exchange zone (UHXZ). Potential influence factors were screened out, and new terms were proposed to quantify the scale of fractures and available heat and cold in the heat sink. Governing equations were established to calculate the impacts of vertical and horizontal fractures on the heat exchange rate in BHX as well as on the heat flow across the UHXZ. The analysis results show that water flow in fractures can significantly enhance heat transfer, reduce required number of boreholes, mitigate heat buildup and reduce irreversibilities underground. The results also suggest that the role of fracture scales and water velocity in GCHP operation should be carefully evaluated. Therefore, detailed hydrogeological survey is necessary. The study results provide a guide on more accurately evaluating the risk of heat buildup and how to take advantage of hydrogeological characters to improve the performance of GCHPs.

## 1. Introduction

**Fig 1:**
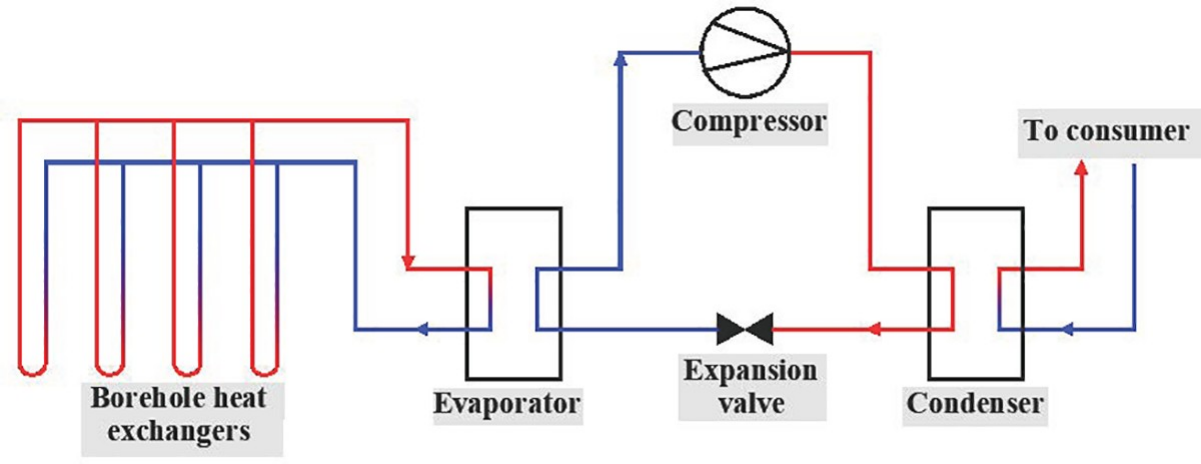
Schematic diagram of GCHP (heating mode).

Shallow geothermal energy is widely distributed and continuous available; furthermore, it offers promising potential in reducing CO_2_ emissions [1–5]. It is recovered and utilized by way of a ground source heat pump (GSHP) [6] which collects or dissipates heat from underground [7]. The ground-coupled heat pump (GCHP) is the most widely applied GSHP (**Fig 1**) and features stabler performance in comparison to an air source heat pump [8]. GCHPs reject heat from buildings into the ground during the summer and collect heat from the ground, which is then transferred to buildings, in the winter [9]. However, the amount of annual heat retrieved from the ground may not be equal to what is initially removed from the ground, resulting in a heat imbalance underground [10]. In most areas, cooling demand is greater than heating demand. Also, heat generated by compressors, fans and pumps can be directly utilized in the heating mode; whereas in the cooling mode, heat generated by those devices must be rejected to the ground [11]. Given these factors, heat imbalance usually appears as heat buildup in the ground.

If the cuboid where the borehole heat exchangers (BHX) are buried is viewed as an underground heat exchange zone (UHXZ), then the reason for heat buildup is due to the inability of heat to be transferred to surrounding formations that are of higher heat capacity. The authors conclude that the phenomenon of heat buildup can in fact be explained as an irreversible loss, or entropy production, in the UHXZ. Some practical approaches to mitigate heat buildup include using a hybrid ground source heat pump system [12], manipulatively constraining heating and cooling loads of GCHP under 15% [13], or increasing distance between heat exchange boreholes [14]. However, implementing these methods results in either an increase in investment or undermines the economics of GCHPs.

On the other hand, fractures and groundwater formations can affect the heat transfer process. Zeng’s test [15] found that the temperatures of two adjacent boreholes at a same depth were substantially different, due to the existence of groundwater flow in a Karst structure. Zeng et al. [15] also concluded that the groundwater table and saturation of soils may be influenced by joints and fractures, which in turn would then impact heat exchange between borehole and soils. Tang [16] evaluated the influence of hydraulic conditions on the performance of the shallow borehole heat exchanger. Hermans et al. [17] concluded that the temperature field of a low-enthalpy geothermal system could be impacted by both heat transfer and water flow, but only physical survey methods were presented. Friberge [18] noted that convection in porous spaces could possess a greater contribution to total heat transfer. Dehkordi et al. [19] and Wang [20] both concluded that groundwater could enhance heat transfer between a borehole and the ground. Yang et al. [21] argued that the presence of groundwater serves a helpful role in mitigating heat imbalance underground. Cui [22] analyzed the heat transfer energy efficiency of buried pipes with variable groundwater temperature.

However, the majority of previous studies focused on the effects of pore water to heat transfer to a single borehole. Few studies, however, have given consideration to the influence of water flow in fractures, and research on the heat transfer between the UHXZ and surrounding formations in fractured rock bodies is insufficient.

Water in fractures is more abundant, has a higher flow rate and fluctuates seasonally, hence it exerts a more evident impact on temperature than porous water. This paper aims to investigate the influence of water flow in fractures on heat buildup and performance of GSHPs. The results demonstrate the major characteristics of fracture water that control the heat transfer mechanism in UHXZ. Fracture water may also prove useful in providing greater accuracy in calculating the heat exchange capacity of boreholes as well as the heat flow between the UHXZ and surrounding formations, evaluating risks of heat buildup, allowing for better site selection and saving unnecessary investment in supplementary facilities.

## 2. Assumptions and methodology

In this study, potential influence factors (scale of fractures, direction of fractures and velocity of groundwater in fractures) were screened out. Next, the study calculated their influences to heat transfer per unit of length of a vertical borehole, total heat flow of a vertical borehole, annual temperature change, irreversibilities and loss of available cold in UHXZ.

### 2.1 General assumption

Without a fracture, heat transfer between vertical boreholes and integrated soil/rocks is dependent on heat conduction. If fracture water exists and directly contacts boreholes, heat transfer is then attributed to heat conduction and heat convection, and the following factors would be impacted:

- Heat flow between a vertical BHX and the ground;
- Heat flow between the UHXZ and surrounding formations;
- Annual heat balance in the UHXZ;
- Annual average temperature variation of the UHXZ; and
- Irreversibilities in the UHXZ.

The groundwater referred to in this study was mainly assumed to be the flowing water in fractures. Consequently, the amount of pore water in the rock matrix is relatively small and its contribution to heat transfer can be ignored compared with the contribution of fracture water. The direction of fractures was assumed to be parallel to the direction of water flow in them. The UHXZ exchange heat with surrounding formations by conduction at its four sides and bottom as well as via convection with water in fractures cutting through it.

Assumptions made in calculation are summarized in **Table 1**. Climate condition of the study case should be introduced. The climate condition of the simulated case is subtropic in southwest China, where cooling demand is higher than heating demand.

**Table 1:** Assumptions in calculation.

Parameters	Assumed value	unit
Buried borehole length, *L*_*b*_	0 ~ 100	m
Formation temperature, *t*_*f*_	288	K
Fracture water temperature, *t*_*gw*_	288	K
Borehole temperature in summer, *t*_*bs*_	303	K
Borehole temperature in winter, *t*_*bw*_	278	K
Heat conductivity of rock, *λ*_*r*_	1.60	W/m·K
Heat conductivity of water, *λ*_*w*_	0.59	W/m·K
Outer radius of the borehole, *r*_*b*_	0.10	m
Temperature change radius, *r*_*tc*_	3.00	m
Space between boreholes, *S*_*b*_	6.00	m
Rock density, *ρ*_*r*_	2,700	kg/m^3^
Specific heat capacity of rock, *Cp*_*r*_	0.80	kJ/(kg.K)
Water velocity range, *u*_*w*_	1–2	mm/s
Thickness of UHXZ, *H*_*g*_	100	m
Cooling demand, *q*_*c*_	1,000	kW
Heating demand, *q*_*h*_	700	kW
Energy Efficiency Ratio of system, *EER*_*sys*_	4.10	
Coefficient of performance of system, *COP*_*sys*_	3.70	
Daily cooling time	24	hr
Daily heating time	24	hr
Cooling period	June, July, August	
Heating Period	December, January, February	

### 2.2 Geometric relationship between fractures and a borehole

**Fig 2:**
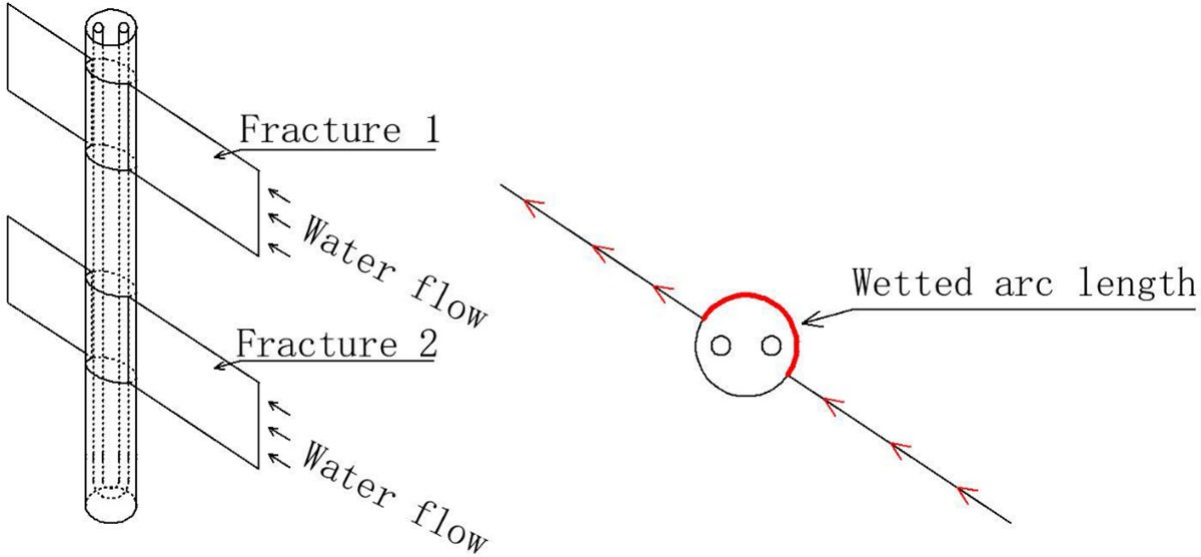
Vertical fractures cross with a borehole.

An analysis was performed on both the geometric relationship between vertical fractures and a borehole and the relationship between horizontal fractures and a borehole. When a section of a vertical borehole is crossed with a vertical fracture (**Fig 2**), a part of the borehole contacts fracture water, and the rest of the borehole contacts rock. When calculating, the term wetting ratio (*W*.*R*.) is defined by the authors as the wetted arc length over the total perimeter of a borehole (**Fig 2**):

$$W.R.=\frac{W.L.}{2\pi r_{b}}$$ (1)

where *W*.*L*. (m) is the wetted arc length on the borehole, and *r*_*b*_ (m) is the out radius of borehole.

When a vertical borehole is crossed with a horizontal fracture, the borehole perimeter is completely wetted by water, and *W*.*R*. is equal to 1.

**Fig 3:**
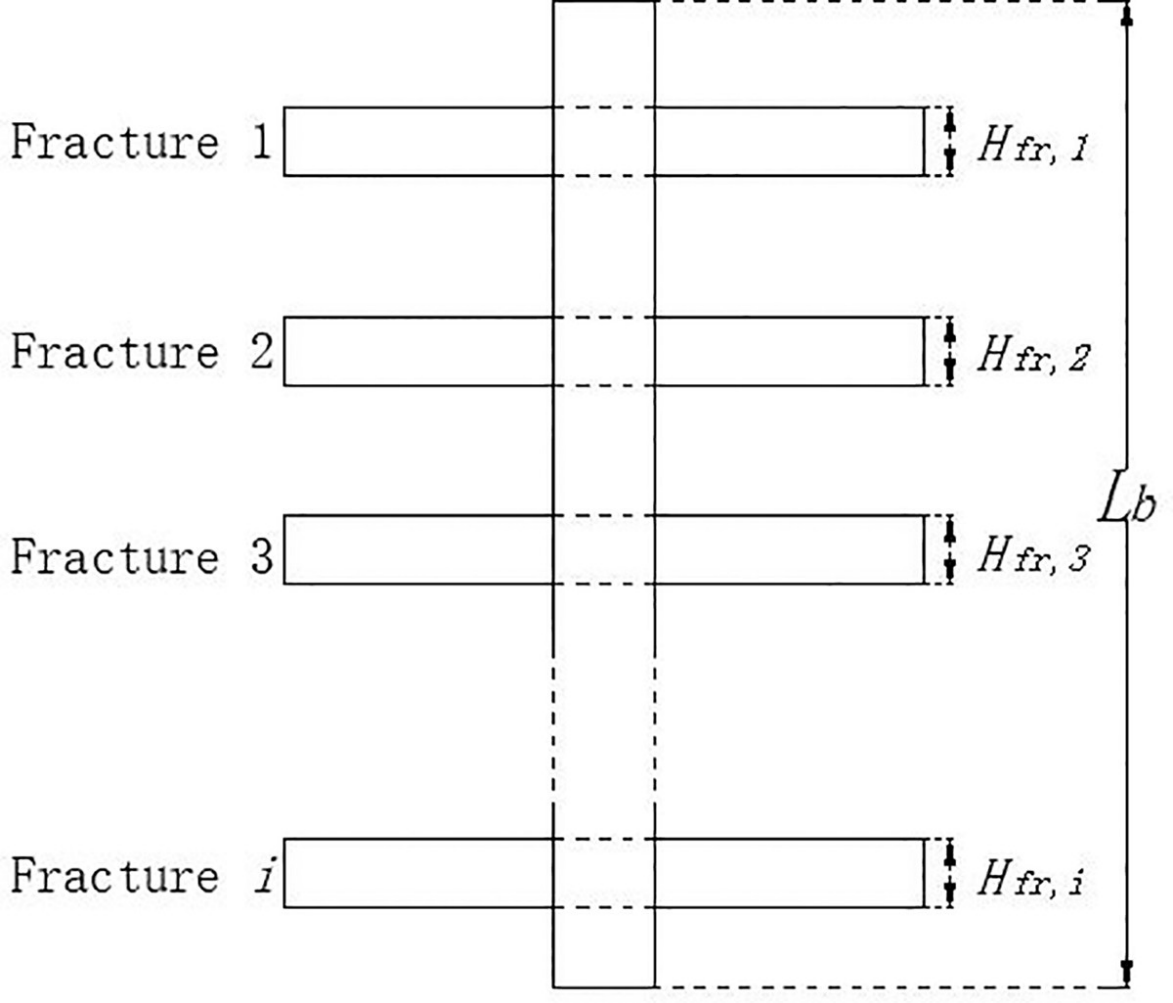
Accumulated height of vertical fracture crossing a borehole.

Assuming that there are *n* (*n*≥1) vertical fractures that cross through a borehole, its heat flow is affected by the total contact area between the fractures and borehole. The total vertical fracture ratio (*Fr*.*V*.*R*.) is introduced by the authors to quantify the scale of vertical fractures, which is defined as the sum of “cross over” heights for each fracture (**Fig 3**) over the length of a borehole:

$$Fr.V.R.=\frac{\sum_{i=1}^{n}H_{fr,i}}{L_{b}}$$ (2)

where *H*_*fr*,*i*_ (m) is the “cross over” height of fracture *i*, and *L*_*b*_ (m) is the length of a vertical borehole buried in the ground (**Fig 3**).

**Fig 4:**
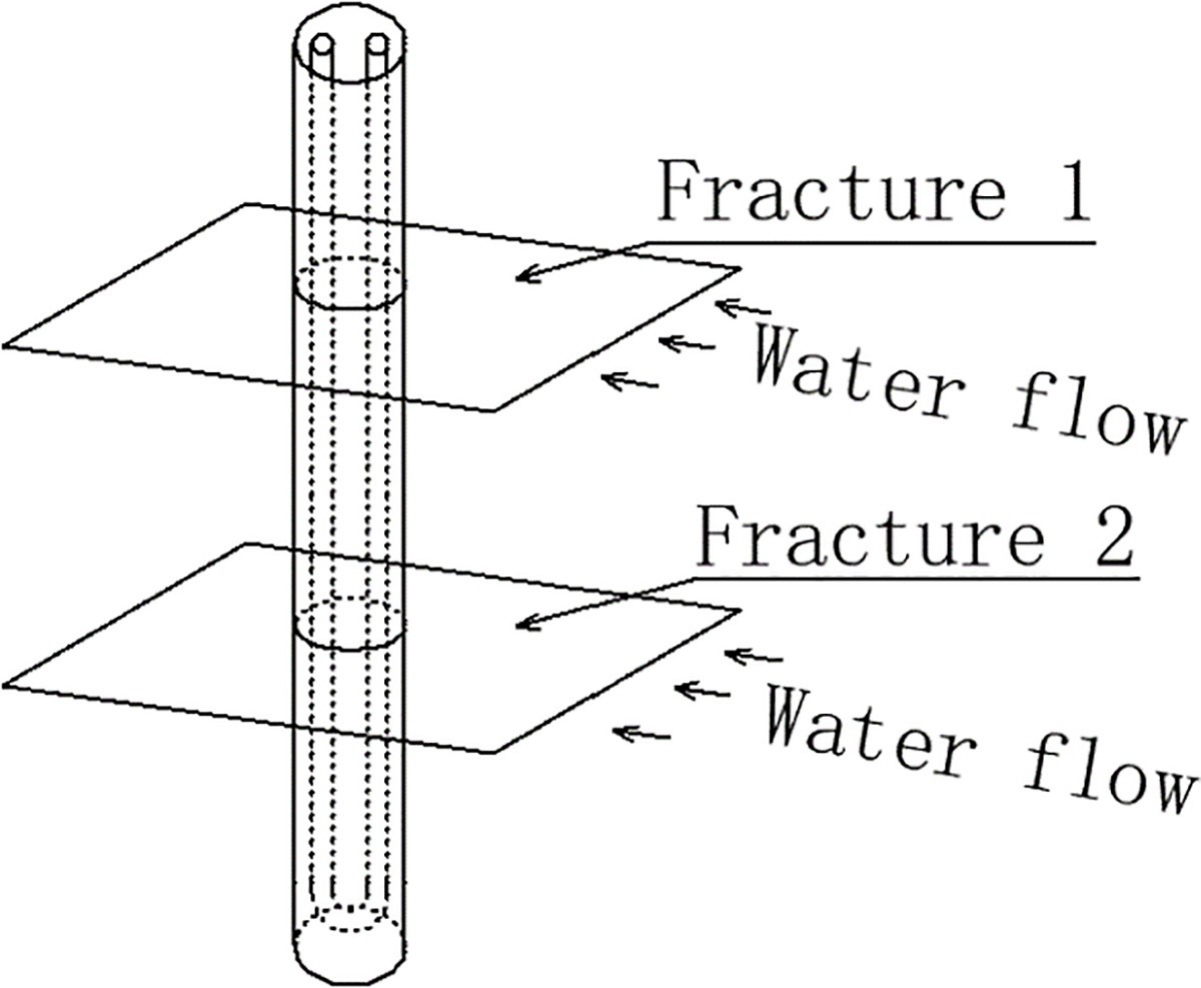
Horizontal fracture crossing a borehole.

**Fig 5:**
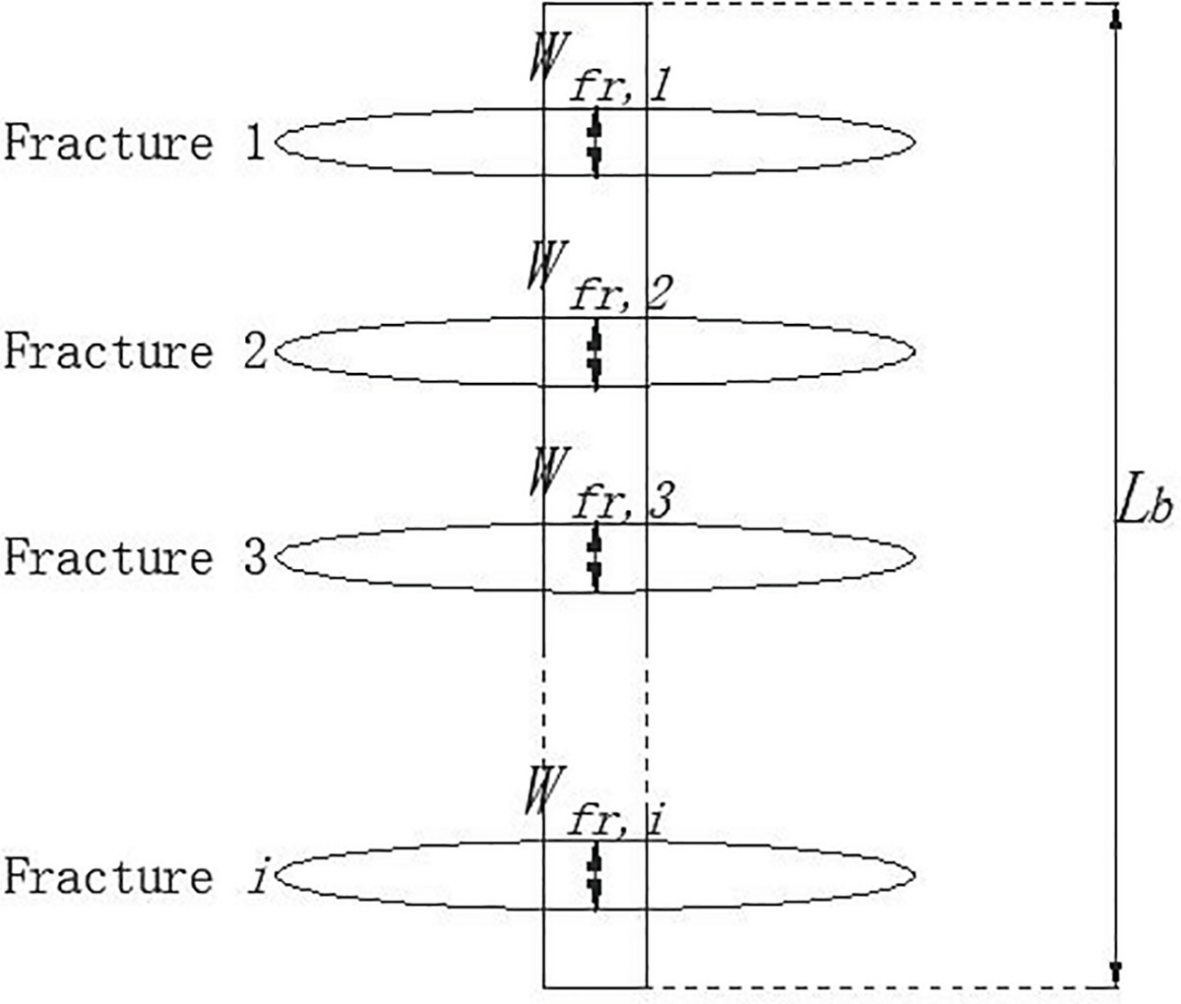
Accumulated width of horizontal fracture crossing a borehole.

Similarly, assuming that there are *n* (*n*≥1) horizontal fractures across a borehole **(Fig 4**), total horizontal fracture ratio (*Fr*._*H*.*R*._) is introduced to quantify the scale of horizontal fractures. *Fr*._*H*.*R*._ is defined as the sum of the “cross over” opening width of each fracture over the length of a buried borehole (**Fig 5**):

$$Fr.H.R.=\frac{\sum_{i=1}^{n}W_{fr,i}}{L_{b}}$$ (3)

where *W*_*fr*,*i*_ (m) is the “cross over” width of fracture *i*, and *L*_*b*_ is the length of a buried borehole.

In the following sections, the heat flow per unit length of borehole and total required number of boreholes were calculated as a function of fracture water velocity, *W*.*R*., *Fr*.*V*.*R*. and *Fr*.*H*.*R*.

### 2.3 Heat flow across UHXZ with fractures

In the summer, heat is rejected and stored in the rock body for later utilization in the winter. In other words, cold is collected from the rock body for the purpose of cooling. Therefore, referring to the Second Law of Thermodynamics and the definition of exergy [23], the term “available cold in rocks” is introduced in this paper. If the temperature of the rock body is lower than the environmental temperature on the surface, *T*_*0*_ (K), it can determine that it contains available cold that could be utilized.

The amount of available cold, *A*.*C*. (kJ), is expressed as:

$$A.C.=m_{r}Cp_{r}\left(T_{0}-T_{c}\right)$$ (4)

where *m*_*r*_ (kg) is the mass of a rock body, *C*_*pr*_ (kJ/kg) is the specific heat capacity of the rock body, and *T*_*c*_ (K) is the temperature of the rock when it is at a relatively cold state compared with the surface environment, and *T*_*c*_ < *T*_*0*_.

Similarly, the amount of available heat in rocks, *A*.*H*. (kJ), is introduced when the rock temperature is higher than the environmental temperature on the surface. This is expressed as:

$$A.H.=m_{r}Cp_{r}\left(T_{h}-T_{0}\right)$$ (5)

where *T*_*h*_ (K) is the temperature of a rock when it is at a relatively warm state compared with the surface environment, and *T*_*h*_ > *T*_*0*_.

The UHXZ undergoes an annual cycle of “heat collect-transit and heat dissipate-transit”. Heat imbalance between extraction and receival would result in loss of *A*.*C*. or *A*.*H*. Thermodynamically, such loss can be explained as being entropy production (*σ*_*cycle*_, kJ/K) or irreversibilities of the cycle as shown in Eq (6) [24]:

$$\oint {\left(\frac{\delta Q}{T}\right)}_{b}=-\sigma_{cycle}$$ (6)

where *δ*_*Q*_ (kJ) represents the heat transfer at a part of the system boundary, and *T* (K) is the absolute temperature at that part of the boundary, which is considered as the formation temperature, *T*_*f*_, in this study.

**Fig 6:**
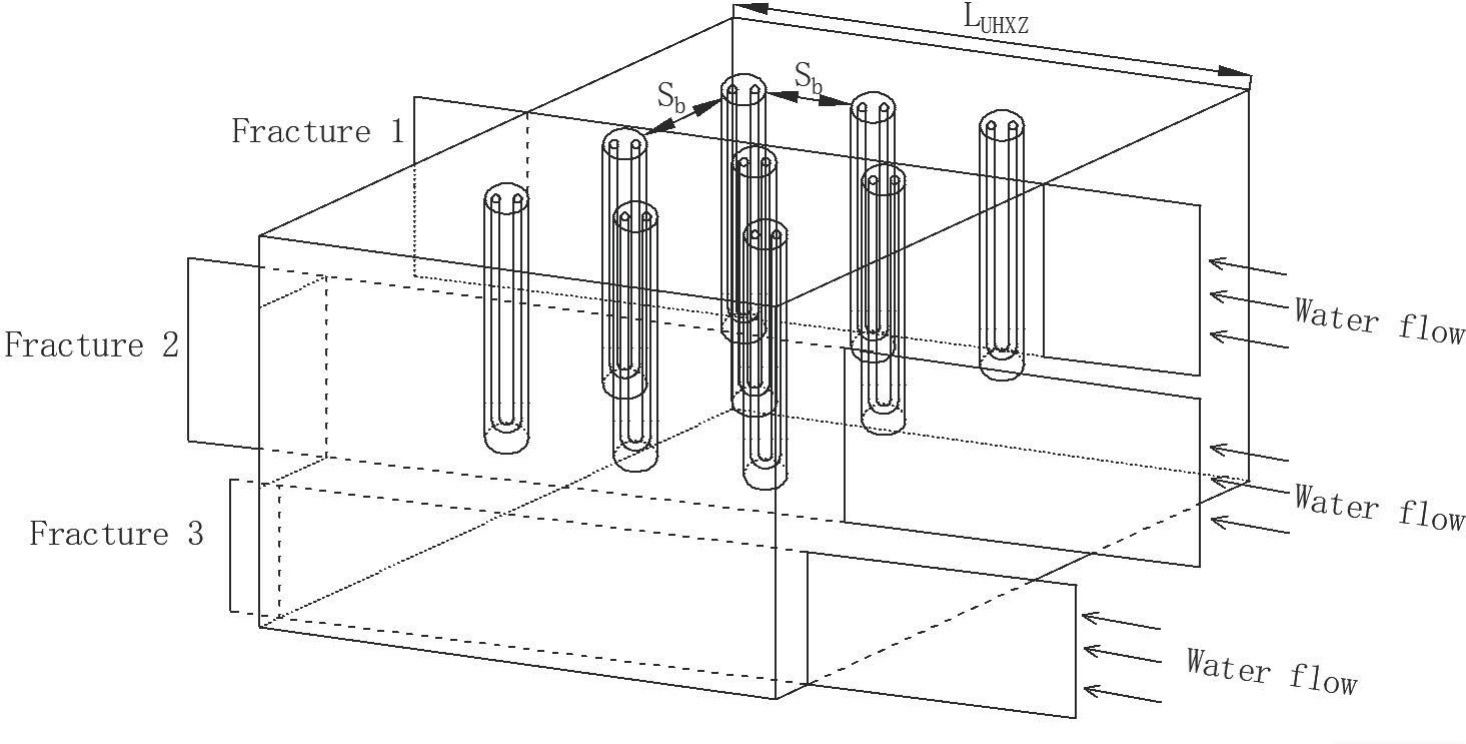
Fractures cross over with the UHXZ.

It is expected that water flow in fractures cutting through the UHXZ would benefit heat exchange between the UHXZ and surrounding formations. Additionally, heat buildup could be mitigated to some extent (**Fig 6**). Potential influence factors include water velocity in fractures and the scale of fractures. The term of total fracture area ratio (*Fr*.*A*.*R*.) is introduced to quantify the scale of fractures. Assuming that the volume of the UHXZ is a square cuboid, *Fr*.*H*.*R*. is defined as the sum of a fracture area inside or on the side of the UHXZ:

$$Fr.A.R.=\frac{\sum_{i=1}^{n}A_{fr,i}}{A_{sUHXZ}}$$ (7)

where *A*_*fr*,*i*_ (m^2^) is the area of fracture *i* in contact with the UHXZ, and *A*_*sUHXZ*_ (m^2^) is the side area of UHXZ, which can be calculated as follows:

$$A_{sUHXZ}=L_{b}\times L_{UHXZ}$$ (8)

where *L*_*UHXZ*_ (m) is the side length of UHXZ, which can be calculated by:

$$L_{UHXZ}=S_{b}\times \sqrt{\frac{q_{re}}{q_{L}\cdot L_{b}}}$$ (9)

where the value of $\sqrt{\frac{q_{re}}{q_{L}\cdot L_{b}}}$ is equal to the number of required boreholes, and *S*_*b*_ (m) represents the spacing between boreholes, which is equal to 2 times *r*_*tc*_.

In the following sections, the annual changes of average temperature, *A*.*C*. and irreversibilities in an UHXZ were calculated as a function of fracture water velocity and *Fr*. *A*.*R*.

## 3. Theoretical fundamentals and equations

### 3.1 Heat transfer between borehole and ground

The overall thermal resistance (*R*_*ov*_, m·K/W) between a borehole and ground at a steady state includes borehole thermal resistance (*R*_*b*_, m·K/W) and ground thermal resistance (*R*_*g*_, m·K/W) [11]:

$$R_{ov}=R_{b}+R_{g}$$ (10)

Existence of fracture water impacts the value of *R*_*g*_ rather than *R*_*b*_; therefore, this study focused on the variation of *R*_*g*_ and assumed *R*_*b*_ to be constant, regardless of the presence of fracture water.

The temperature in the rock around the borehole, *T*_*R*_ (K), from a specific distance from the center of borehole, *r* (m), is in the form of axial symmetry and determined by Eq (11) [25]:

$$T_{r}=T_{b}^{}-\left(T_{b}-T_{f}\right)\frac{\ln\left(\frac{r}{r_{tc}}\right)}{\ln\left(\frac{r_{tc}}{r_{b}}\right)}$$ (11)

where *T*_*b*_ (K) is the temperature of the borehole and assumed to be constant in this study, *T*_*f*_ (K) is the temperature of the geological formation, and *r*_*tc*_ (m) is the temperature change radius, which is measured from the center of the borehole and defined as being beyond the point where the temperature disturbance from the borehole can be ignored.

When there is no fracture water present nor pure heat conduction between the borehole and rock, heat flow per meter of the borehole, *q*_*l*,*cd*_ (w/m) is calculated by [25]:

$$q_{l,cd}=\frac{T_{b}-T_{f}}{\frac{1}{2\pi \lambda_{r}}\ln\left(\frac{r_{tc}}{r_{b}}\right)}$$ (12)

where *λ*_*r*_ (W/m·K) is the heat conductivity of the rock.

The corresponding thermal resistance of conduction between the borehole and the rock, *R*_*g*,*cd*_ (m·K/W), is:

$$R_{g,cd}=\frac{1}{2\pi \lambda_{r}}\ln\left(\frac{r_{tc}}{r_{b}}\right)$$ (13)

If there is fracture water and the BHX is immersed in it, heat transfer would be completely dependent on heat convection, and the heat flow of the unit length between borehole and water, *q*_*l*,*cv*_ (w/m), would be calculated by [25]:

$$q_{l,cv}=2\pi r_{b}h(T_{b}-T_{gw})$$ (14)

where *h* (W/m^2^·K) is the heat convection coefficient, *T*_*gw*_ (K) is the temperature of groundwater in fractures.

The corresponding thermal resistance of convection between borehole and rock, *R*_*g*,*cv*_ (m·K/W), is:

$$R_{g,cv}=\frac{1}{2\pi r_{b}h}$$ (15)

Since the borehole spacing is much larger than its diameter, the borehole is simulated as a single cylinder in cross flow, with the value of *h* is calculated as the following [26]:

:

$$h=Nu\cdot \frac{\lambda_{W}}{2r_{b}}$$ (16)

where λ_w_ (W/m·K) is the heat conductivity of water which is determined by the state of water, *Nu* is the Nusselt number. The value is *Nu* is calculated by the Churchill–Bernstein equation [26]:

$$Nu=0.3+\frac{0.62Re^{\frac{1}{2}}Pr^{\frac{1}{3}}}{{\left[1+{\left(\frac{0.4}{Pr}\right)}^{\frac{2}{3}}\right]}^{\frac{1}{4}}}{\left[1+{\left(\frac{Re}{282000}\right)}^{\frac{5}{8}}\right]}^{\frac{4}{5}}$$ (17)

where *Re* is the Reynolds number, and *Pr* is the Prandtl number determined by the state of the water.

The *Re* is calculated by:

$$Re={\frac{u_{w}d_{b}}{\nu_{w}}}^{}$$ (18)

where *u*_*w*_ (m/s) is the velocity of water, *d*_*b*_ (m) is the diameter of heat exchange borehole, and *ν*_*w*_ (m^2^/s) is the dynamic viscosity of water.

In the case of only a part of the borehole contacting with water, the total heat flow per meter of a borehole (*q*_*l*_, W/m) is contributed by both heat conduction and convection, and is calculated by:

$$q_{l}=(1-W.R.)\frac{T_{b}-T_{f}}{\frac{1}{2\pi \lambda_{r}}\ln\left(\frac{r_{tc}}{r_{b}}\right)}+W.R.\cdot 2\pi r_{b}h\left(T_{b}-T_{gw}\right)$$ (19)

During a cooling period in the summer, compressors, fans and pumps generate extra heat rejected to the ground, and the heat rejection rate to ground, *q*_*re*_ (W), is calculated by the following equation [11]:

$$q_{re}=(1+\frac{3.412}{EER_{sys}})\times q_{c}$$ (20)

where *EER*_sys_ (Btu/Wh) is the Energy Efficiency Ratio of the heat pump system [27], and *q*_*c*_ (W), is the cooling demand.

During a heating period, heat generated by those devices can be directly utilized, and the heat extraction rate from the ground, *q*_*ex*_ (W), is calculated by:

$$q_{ex}=(1-\frac{1}{COP_{sys}})\times q_{h}$$ (21)

where *COP*_*sys*_ is the Coefficient of performance for the heat pump system [27], and *q*_*h*_ (W), is the heating demand.

### 3.2 Heat transfer in the *UHXZ*

The *UHXZ* also exchanges heat with surrounding rocks through heat conduction and with ground water flow through convection.

During the cooling season, the heat exchange rate *q*_*c*_ (W) from the UHXZ to surrounding rocks through conduction is calculated by:

$$q_{c}=\left(L_{UHXZ}^{2}+4\cdot H_{g}\cdot L_{UHXZ}\right)\cdot \lambda_{r}\cdot \left(T_{UHXZ,C}-T_{f}\right)$$ (22)

where *H*_*g*_ (m) is the thickness of the UHXZ, *L*_*UHXZ*_ (m) is the equivalent side length of the UHXZ, and *T*_*UHXZ*,*C*_ (K) is the temperature of the UHXZ in the cooling season.

Similarly, during the heating season, the heat exchange rate *q*_*h*_ (W) from the UHXZ to surrounding rocks by means of conduction is calculated by:

$$q_{h}=\left(L_{UHXZ}^{2}+4\cdot H_{g}\cdot L_{UHXZ}\right)\cdot \lambda_{r}\cdot \left(T_{f}-T_{UHXZ,h}\right)$$ (23)

where *T*_*UHXZ*,*h*_ (K) is the temperature of the UHXZ in the heating season.

During the cooling season, heat exchange rate *q*_*c*,*w*_ (W) from the UHXZ to ground water flow in fractures through convection is calculated by:

$$q_{c,w}=A_{fr}\cdot h_{fr}\cdot \left(T_{UHXZ,C}-T_{gf}\right)$$ (24)

where *A*_*fr*_ is the area of fracture cutting through the UHXZ, and *h*_*fr*_ (W/m^2^∙K) is the convective coefficient between rock and water flow.

The fracture is viewed as a tube with a rectangular cross-section, and its *h*_*fr*_ is calculated by [26]:

$$h_{fr}=Nu_{fr}\cdot \frac{\lambda_{W}}{d_{fr}}$$ (25)

where *d*_*fr*_ is the equivalent diameter of the fracture tube, and *Nu*_*fr*_ is the Nusselt number which is calculated by the Dittus-Boelter Equation [26]:

$$Nu_{fr}=0.023Re_{fr}{}^{0.8}Pr^{0.3}$$ (26)

where *Re*_*fr*_ is the Reynolds number of flow in the fracture, which is given by:

$$Re_{fr}={\frac{u_{w}d_{fr}}{\nu_{w}}}^{}$$ (27)

Similarly, during the heating season, the heat exchange rate *q*_*h*,*w*_ (W) between the UHXZ and ground water flow through convection is calculated by:

$$q_{h,w}=A_{fr}\cdot h_{fr}\cdot \left(T_{gf}-T_{UHXZ,h}\right)$$ (28)

The annual change of formation temperature of UHXZ, *ΔT*_*f*,*a*_, is calculated by:

$$\Delta T_{f,a}=\frac{\left(q_{re}+q_{h}+q_{h,w}-q_{ex}^{}-q_{c}-q_{c,w}\right)\cdot 3600\cdot 24\cdot 30\cdot 3}{Cp_{r}\cdot \rho_{r}\cdot H_{g}\cdot L_{UHXZ}^{2}}$$ (29)

where *Cp*_*r*_ (kJ/(kg∙K)) is the specific capacity of rock, *ρ*_*r*_ (kg/m^3^) is the density of rock, 3 (months), 30 (days), 24(hours), and 3600 (seconds) are conversion constants for working duration, and *L*_*UHXZ*_ is the side length of UHXZ when its projected area is assumed to be square and is calculated by:

$$L_{UHXZ}=(R_{b}-1)\cdot S_{b}$$ (30)

where *S*_*b*_ (m) is the space between boreholes, *N*_*rb*_ is the number of rows of boreholes, which is calculated by:

$$N_{r}{}_{b}=\sqrt{\frac{\max(L_{bh},L_{bc})}{H_{g}}}$$ (31)

where *L*_*bh*_ (m) is the total required length of boreholes meeting the heating demand, and *L*_*bc*_ (m) is the total required length of boreholes meeting the heating demand, all of which is calculated as:

$$L_{bh}=\frac{q_{re}}{q_{l}}$$ (32)

$$L_{bc}=\frac{q_{ex}}{q_{l}}$$ (33)

## 4. Results analysis

### 4.1 Impacts of vertical fractures to the heat flow of boreholes

**Fig 7:**
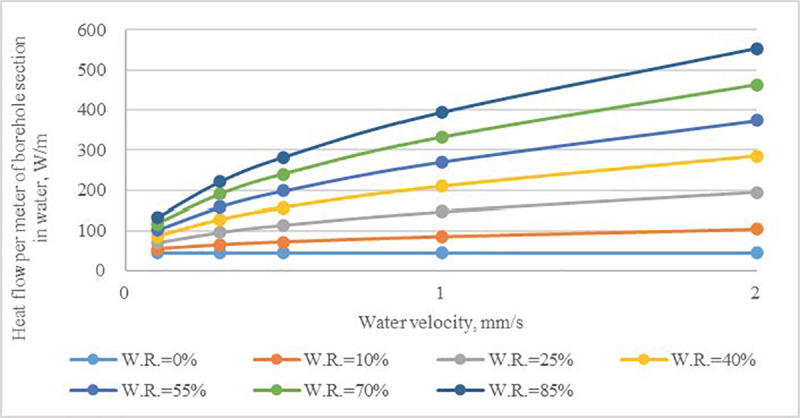
Heat flow rate per meter of borehole section in contact with water flow as a function of water velocity and value of *W*.*R*.

From the governing equations, heat convection between a borehole and fracture water is related to water velocity and the extent of being wetted, which can be expressed by the value of *W*.*R*. Therefore, for the borehole’s partial section that contacts with water, the heat flow per meter as a function of *W*.*R*. and water velocity were calculated (**Fig 7**). Heat transfer was significantly enhanced by convection with water flow in fractures. The heat flow per meter increased with both *W*.*R*. and water velocity, and the gradient of curves became more pronounced when the *W*.*R*. increased.

**Fig 8:**
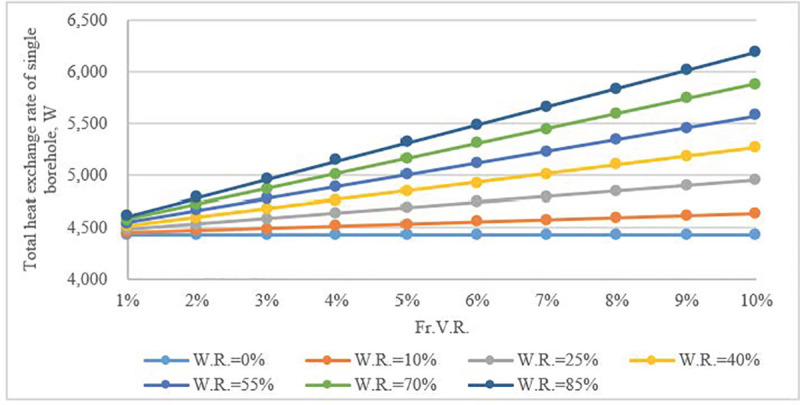
Total heat flow rate of single borehole as a function of *Fr*.*V*.*R*. and *W*.*R*., fracture water velocity = 0.0005 mm/s.

**Fig 9:**
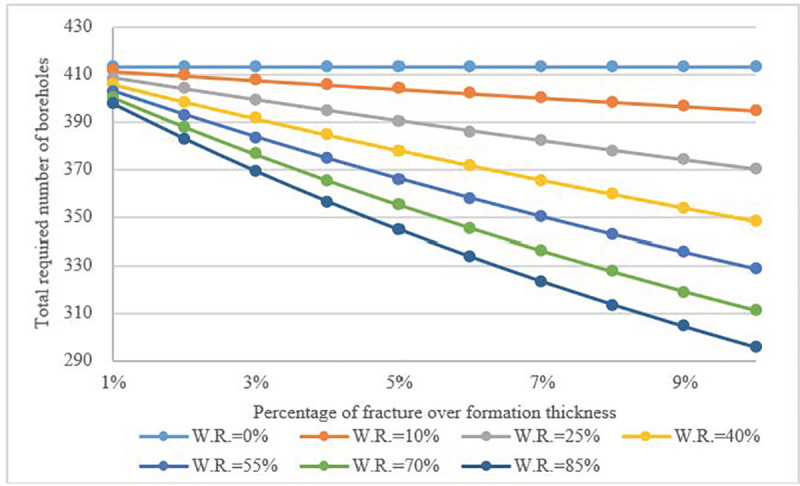
Required number of boreholes as a function of *Fr*.*V*.*R*. and *W*.*R*., fracture water velocity = 0.0005 mm/s.

The impact of scale of vertical fractures was further investigated when the water flow velocity was equal to 0.0005 mm/s. The calculation result in (**Fig 8**) demonstrates that, due to the impact from the flow in fractures, the total heat transfer rate of a single borehole improved significantly. The influence of *Fr*.*V*.*R*. was more obvious than that of *W*.*R*. As the heat exchange capacity increased, the required number of boreholes and investment decreased (**Fig 9**). The required number of boreholes could be reduced by up to 30% compared to when a borehole is not wetted.

### 4.2 Impact of horizontal fractures to the heat flow in a borehole

**Fig 10:**
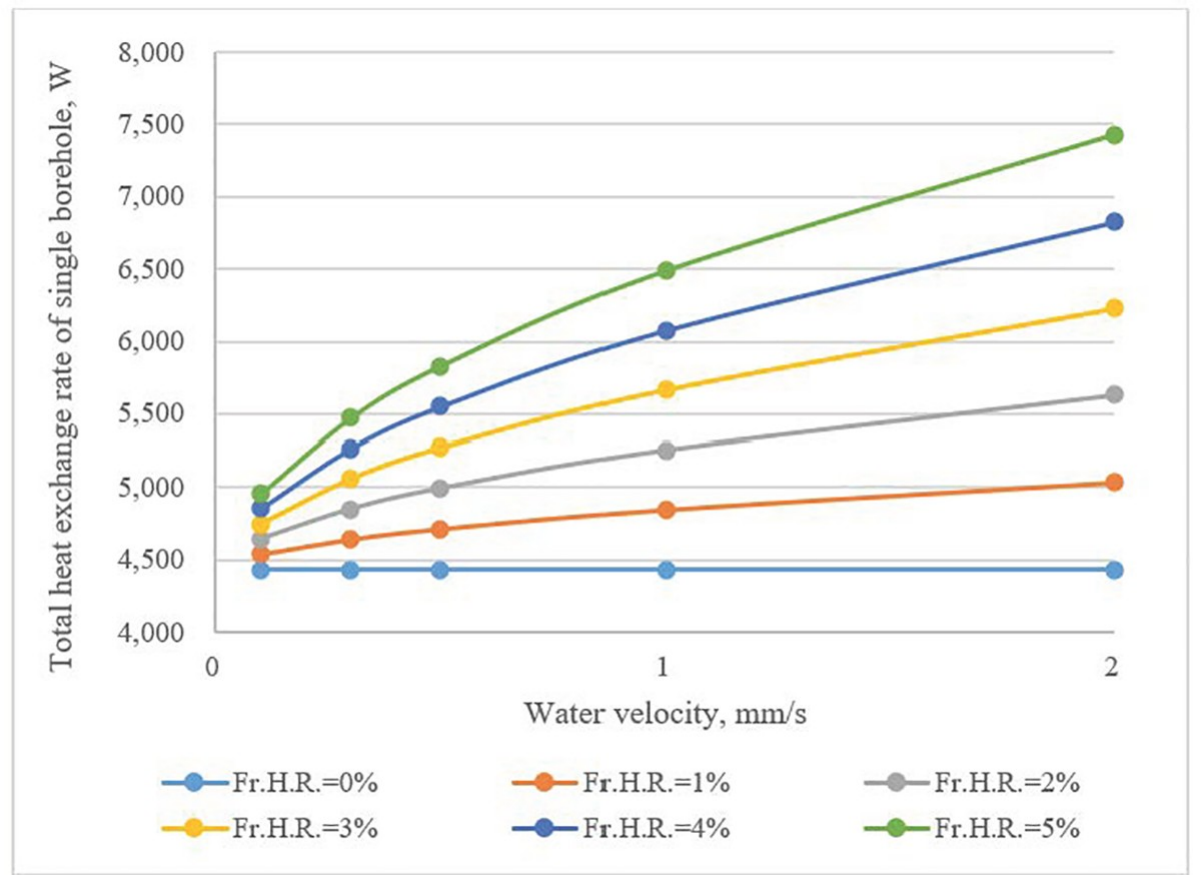
Heat flow per meter of borehole as a function of water velocity in horizontal fractures and *Fr*.*H*.*R*.

**Fig 11:**
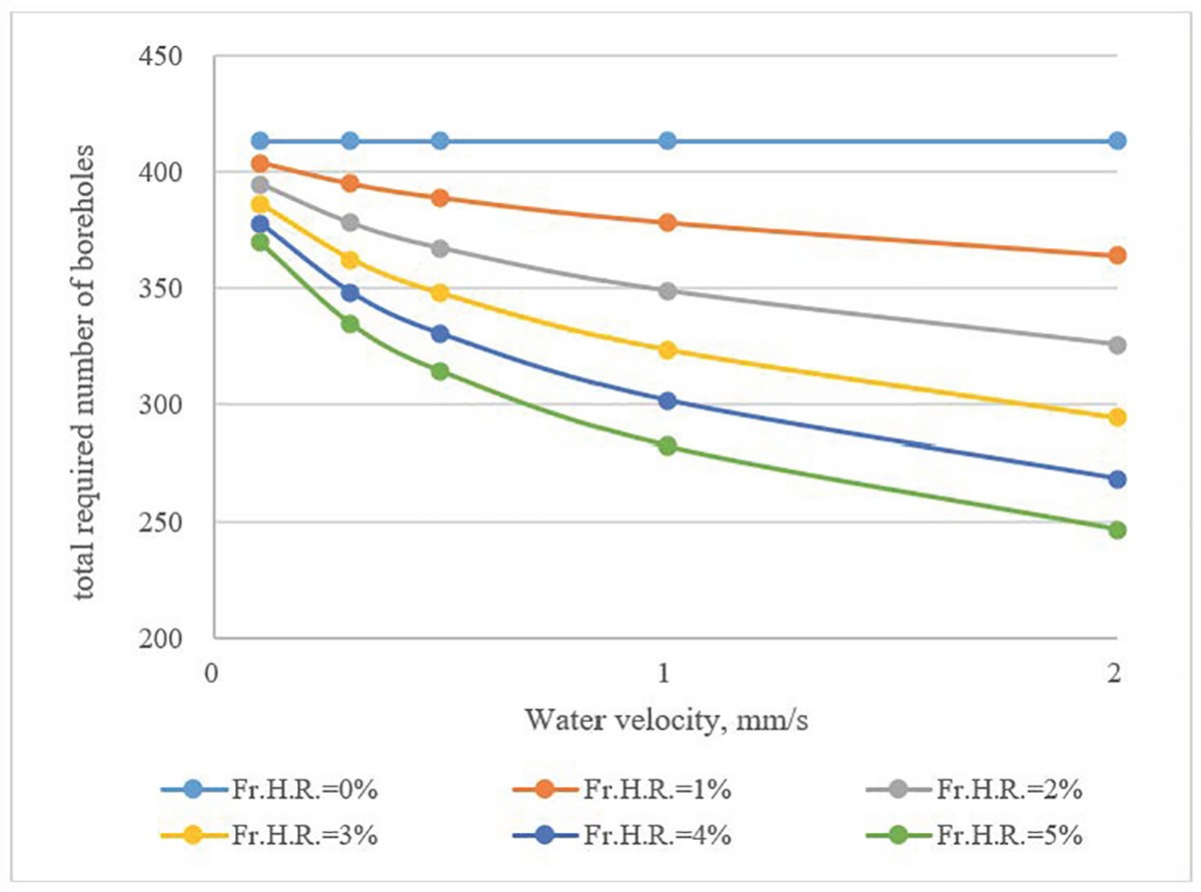
Required number of boreholes as a function of *Fr*. *H*.*R*. and fracture water velocity.

The calculation results in (**Fig 10**) indicate that the total heat exchange rate of a borehole was proportional to water velocity in fractures, and its influence became more obvious with a high value of *Fr*.*H*.*R*. The number of boreholes can be reduced by as much as 40% compared to cases that featured no water flow (**Fig 11**).

### 4.3 Impacts of fractures on the UHXZ

**Fig 12:**
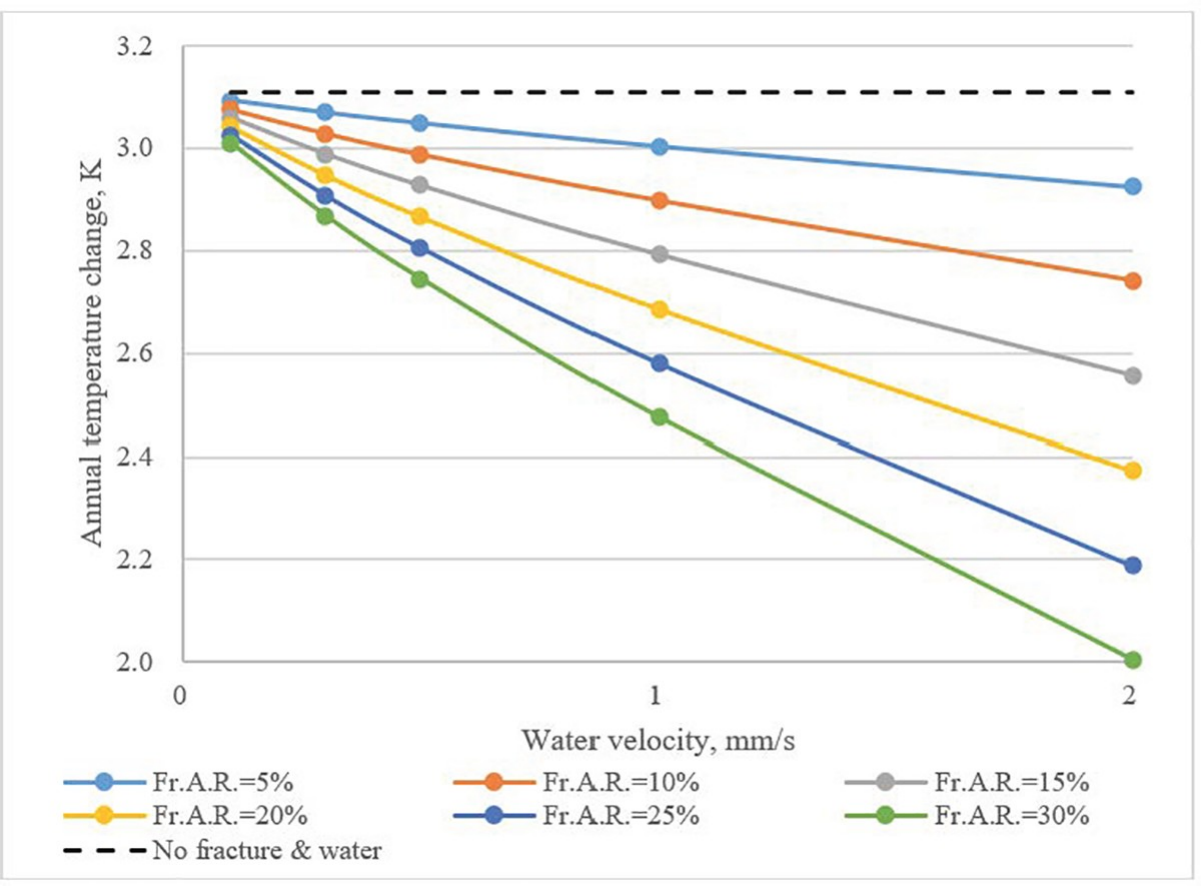
Annual temperature change of UHXZ as a function of water velocity and *Fr*.*A*.*R*.

The calculated annual change of average temperature in the UHXZ as a function of *Fr*.*A*.*R*. and water velocity can be seen in **Fig 12**. When there was no fracture water, the average temperature in the UHXZ increased by approximately 3.1 K annually. The temperature variation fell when groundwater flow in fractures were present. As the *Fr*.*A*.*R*. increased, the influence from water velocity became more obvious. Heat buildup could be mitigated by the impacts of fracture water flow.

**Fig 13:**
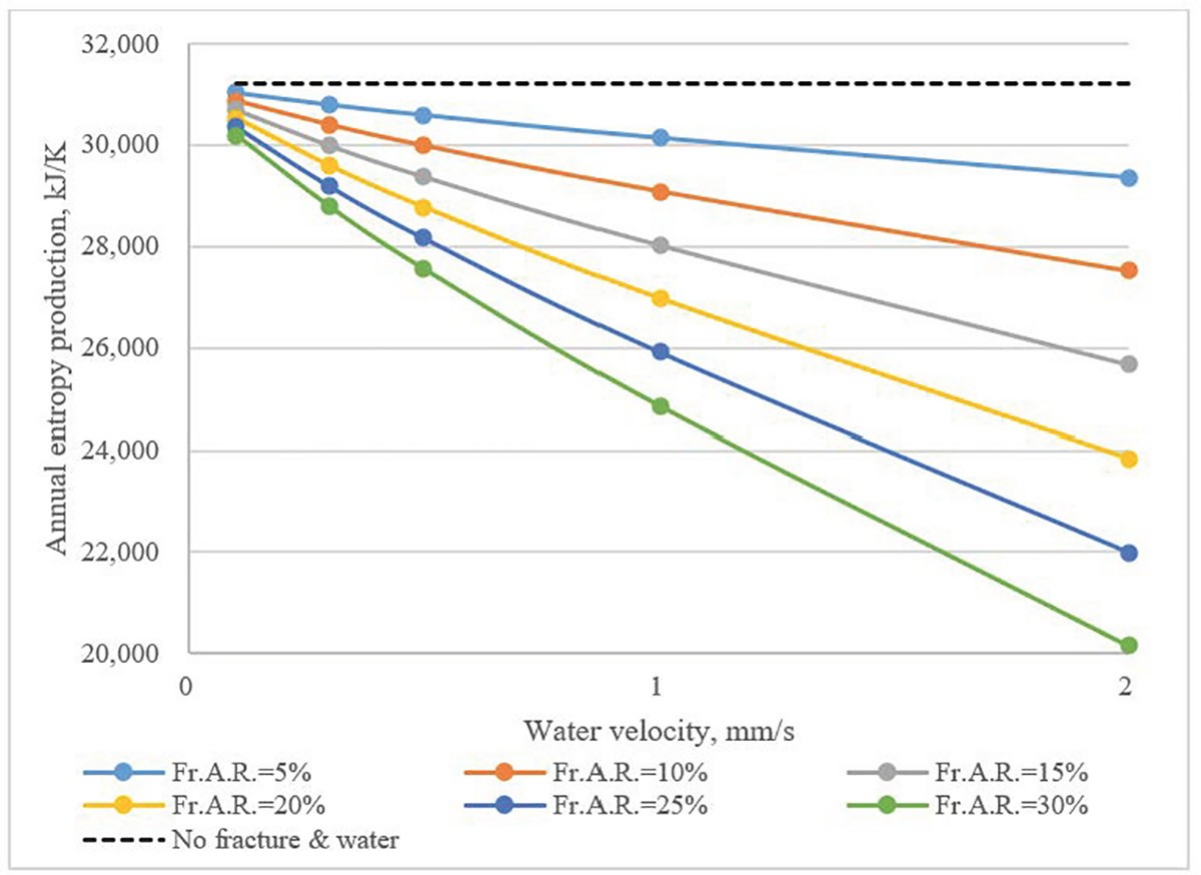
Total annual irreversibilities (entropy production) of UHXZ as a function of water velocity and *Fr*.*A*.*R*.

The same trend can be also seen in **Fig 13**, which shows the overall variation of irreversibilities (entropy production) in the UHXZ over an annual operation cycle. The irreversibilities were significantly reduced with the presence of fracture water flow.

**Fig 14:**
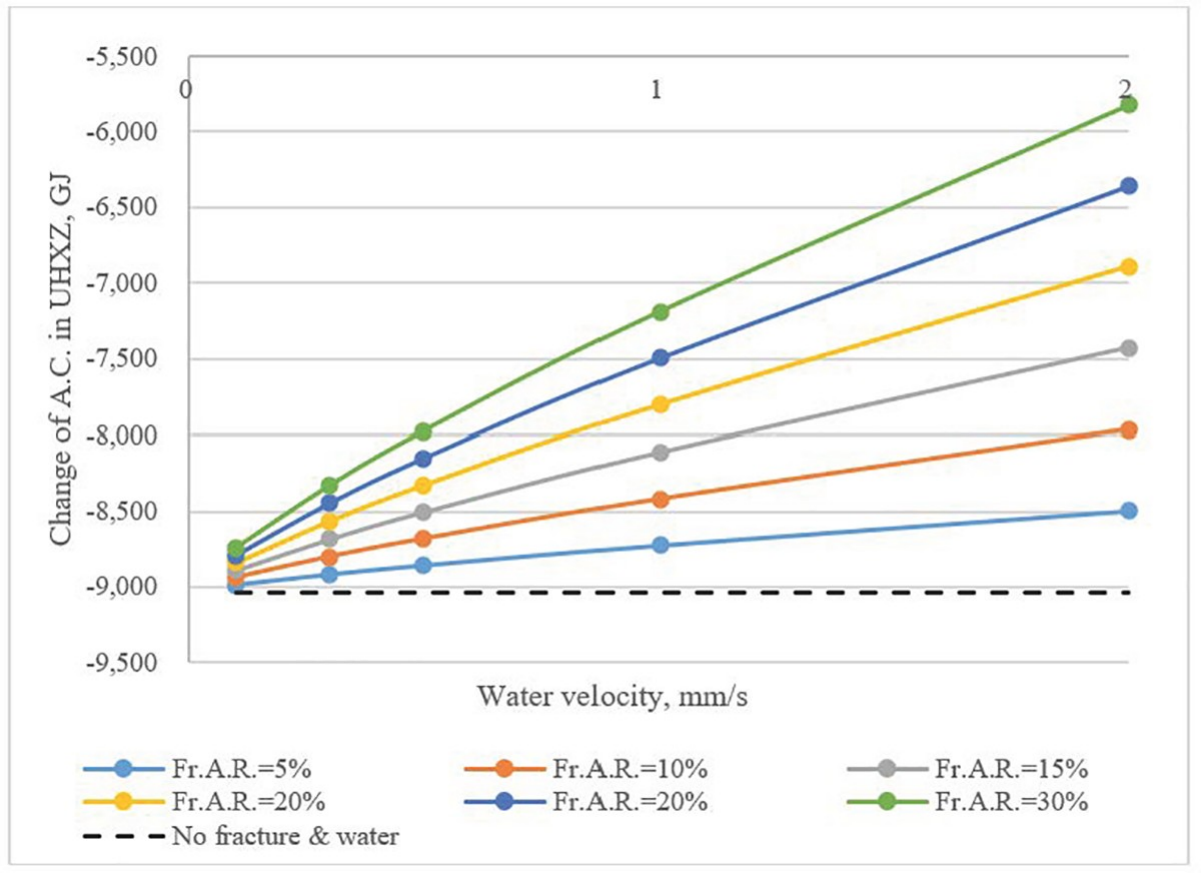
Annual change of *A*.*C*. in UHXZ as a function of water velocity and *Fr*.*A*.*R*.

The loss of available cold in the UHXZ is shown in **Fig 14**. Since heat storage capability of the rock body was enhanced with the presence of fracture water flow, a lower amount of available cold was lost in the annual cycle. The *Fr*.*A*.*R*. had a more pronounced influence than water flow velocity.

## 5. Discussion

From the results of the calculations above, it can be seen that the presence of water flow in fractures can significantly enhance the heat exchange capacity of single borehole as well as heat transfer between the UHXZ and surrounding formations. Thus, water flow in fractures play a role in mitigating irreversibilities and heat buildup in the UHXZ.

For the section of a borehole crossed by vertical fractures, there was a clear increase in the heat flow rate when the water velocity was at a low range (< 0.05 mm/s). Both *Fr*.*V*.*R*. and *W*.*R*. had a pronounced effect on the heat flow rate of a borehole, and the effect of *Fr*.*V*.*R*. became increasingly evident with the rise of *W*.*R*. As the total heat flow rate of single borehole increased, the required number of boreholes dropped, which could allow for associated investments to be saved.

For a borehole crossed with horizontal fractures, the *Fr*.*H*.*R*. had a more pronounced impact than water velocity on the heat flow rate per meter of a borehole. It is worth noting that the impact of water velocity increased with the value of *Fr*.*H*.*R*. The decrease required number of boreholes was also obvious.

Water flow in fractures also enhanced heat transfer between the UHXZ and surrounding formations. In an annual cycle, heat buildup, irreversibilities and loss of available cold in the UHXZ were significantly mitigated. When there was no fracture water, the annual elevation of the average temperature reached 3.1 K. With the presence of fracture water flow, the elevation of the average temperature was reduced to 2.0K. In comparison to water velocity, *Fr*.*A*.*R*. had a more obvious effect. This effect is even more evident when considering that previous studies [28] showed the increment of soil temperature, with the seepage of pore water, could be narrowed by 1.5 K. Therefore, the beneficial effect of fracture water in mitigating heat buildup is clear.

In the discussions above, *Fr*.*V*.*R*., *Fr*.*H*.*R*. and *Fr*.*A*.*R*. served as quantified indicators to represent the scale of fractures. It can be seen that, in general, the scale of fractures had a more pronounced influence than the velocity of water flow in fractures.

## 6. Conclusion

Heat buildup is a major issue impeding further improvement and popularization of GCHPs. This is caused by additional heat being rejected to the ground that cannot be transferred from the UHXZ to surrounding formations that have a greater heat capacity. This results in irreversibilities and the loss of available cold in the UHXZ. Previous studies focused on the influences of pore water on the heat transfer of a single borehole, but there presently is an insufficient understanding of the effect of fracture water and heat transfer across the boundary of UHXZ. Current engineering practices resort to high cost solutions to mitigate heat buildup.

In this paper, the effects of water flow in fractures on the heat flow rate of a single borehole and heat exchange between the UHXZ and surrounding formations were investigated. The concepts of “available cold”, “available heat” and quantified indicators used to represent the scale of fractures were introduced. The temperature change and irreversibilities in the UHXZ over an annual operation cycle were calculated. It can be concluded that the existence of fracture water flow is obviously beneficial in terms of enhancing the heat exchange rate for a single borehole and the UHXZ. Therefore, the required number of boreholes and associated investment can be reduced, and heat buildup is subsequently mitigated. In general, the scale of fractures has a more pronounced influence than that of water velocity.

The study results are useful for site selection, more accurate estimation of heat transfer capacity underground, and the evaluations into the risk of heat buildup. It is suggested that, for engineering projects, detailed geological surveys on hydrology and fracture development should be performed. If construction requirements permit, it is suggested that sites with developed fractures and groundwater source be given preference, since these aforementioned advantages can be taken to improve techno-economics of GSHP systems, as opposed to relying on other solutions that increase investment costs or undermine the economics of the system.

## Abbreviations

***Acronyms*** BHX: borehole heat exchanger
GSHP: ground source heat pump
GCHP: ground-coupled heat pump
UHXZ: underground heat exchange zone
***Units*** Btu: British thermal unit
hr: hour
K: Kelvin degree
kg: kilogram
kJ: kilojoule
m: meter
m^2^: square meter
m^3^: cubic meter
s: second
W: watt
Wh: watt-hour
***Symbols*** A.C.: available cold (kJ)
A.H.: available heat (kJ)
A_fr,i_: area of fracture i contacting with the UHXZ (m^2^)
A_fr_: area of fracture cutting through the UHXZ (m^2^)
A_sUHXZ_: side area of UHXZ (m^2^)
COP_sys_: coefficient of performance of the heat pump system
Cp_r_: specific heat capacity of rock body (kJ/(kg∙K))
d_b_: diameter of heat exchange borehole (m)
d_fr_: equivalent diameter of the fracture tube (m)
EER_sys_: Energy Efficiency Ratio of the heat pump system (Btu/Wh)
Fr.A.R.: fracture area ratio
Fr.H.R.: total horizontal fracture ratio
Fr.V.R.: total vertical fracture ratio
h: heat convection coefficient (W/m^2^∙K)
H_fr,i_: “cross over” height of fracture i (m)
h_fr_: convective coefficient between rock and water flow (W/m^2^∙K)
H_g_: thickness of the UHXZ (m)
L_b_: length of a vertical borehole exchange in ground (m)
L_bc_: total required length of boreholes meeting the cooling demand (m)
L_bh_: total required length of boreholes meeting the heating demand (m)
L_UHXZ_: equivalent side length of the UHXZ (m)
m_r_: mass of rock body (kg)
N_rb_: number of rows of boreholes
Nu: Nusselt number
Pr: Prandtl number
q_c_: cooling demand (W)
q_ex_: heat extraction rate from ground (W)
q_h_: heating demand (W)
q_h,w_: heat exchange rate(W)
q_l_: total heat flow per meter of borehole (W/m)
q_l,cd_: heat flow per meter of borehole by conduction (W/m)
q_l,cv_: heat flow of unit length between borehole and water (W/m)
q_re_: heat rejection rate to ground (W)
q_c,w_: heat exchange rate
r: distance from the center of borehole (m)
rb: out radius of borehole (m)
R_b_: borehole thermal resistance (m∙K/W)
Re: Reynolds number
Re_fr_: Reynolds number of flow in the fracture
R_g_: ground thermal resistance (m∙K/W)
R_g,cd_: thermal resistance of conduction between borehole and rock (m∙K/W)
R_g,cv_: thermal resistance of convection between borehole and rock (m∙K/W)
R_ov_: overall thermal resistance (m∙K/W)
r_tc_: temperature change radius (m)
S_b_: spacing between boreholes (m)
T: temperature of boundary (K)
T_0_: environment temperature on the surface (K)
T_b_: temperature of the borehole (K)
T_c_: temperature of rock at a relatively cold state (K)
T_f_: temperature of geological formation (K)
T_gw_: temperature of groundwater in fractures (K)
T_h_: temperature of rock at a relatively warm state (K)
T_R_: temperature in the rock around the borehole (K)
T_UHXZ,C_: temperature of the UHXZ in cooling season (K)
T_UHXZ,h_: temperature of the UHXZ in heating season (K)
u_w_: velocity of water (m/s)
ν_w_: dynamic viscosity of water
W.L.: wetted arc length (m)
W.R.: wetting ratio
W_fr,i_: “cross over” width of fracture i (m)
x: length of fracture (m)
δQ: heat transfer at boundary (kJ)
λ_r_: heat conductivity of rock (W/m.K)
λ_w_: heat conductivity of water (W/m.K)
ν_w_: dynamic viscosity of water (m^2^/s)
σ_cycle_: entropy production (kJ/K)
ΔT_f,a_: annual change of formation temperature of UHXZ (K)
ρ_r_: density of rock (kg/m^3^)
